# King Edward's Hospital Fund for London

**Published:** 1905-01-07

**Authors:** 


### HOSPITAL ADMINISTRATION.

### CONSTRUCTION AND ECONOMICS.

### KING EDWARD'S HOSPITAL FUND FOR LONDON.

### A MUNIFICENT NEW YEAR'S GIFT.

### THE KING'S THANKS.

LORD MOUNT STEPHEN has truly declared that his gift of securities for £200,000, yielding £11,000 a year, affords an opportunity for some one, both able and willing, to do a beneficent act in aid of the most deserving of all our charities by contributing securities to an amount which will yield £3,000 a year more. If this is done promptly the permanent income of the King's Fund from invested funds will amount to £50,000 a year, so that the total sum available for distribution through its agency is never likely to be less than £100,000 a year in future. The King, who is ever ready to recognise and appreciate loyal service unselfishly rendered in any noble cause, in his autograph letter of thanks to Lord Mount Stephen, expressing his high appreciation of the gift, has well described it as a magnificent donation.

Lord Mount Stephen has been noted throughout his career for the magnificence of his gifts to charity. He gave, in conjunction with Lord Strathcona, a splendid new hospital to Montreal. The same two noble benefactors in 1902 handed over securities to the value of £400,000 to the King's Fund. They have lived so much amongst us, and have so fully entered into our national life, that the public may scarcely realise that the fortunes from which these gifts spring were made in the Dominion of Canada, and that the gifts to the King's Fund typify the binding force resulting from a united Empire. It would be difficult to exaggerate the value and importance of these magnificent contributions to the London hospitals through the King's Fund. They reflect the greatest honour upon the givers; they indicate that an enormous fortune, built up in the life of any one man, is held nowadays by the best and most intelligent members of our nation to be, after all, but a great trust, as it is also a great responsibility, and that the possession of vast wealth entails upon the possessor responsibilities which Lord Mount Stephen and Lord Strathcona have so nobly lived up to. The force of example is great, and we anticipate that the indirect effects of this splendid munificence may, in the near future, produce results as important as those directly accruing to the King's Fund from the gifts themselves.

---

THE following letter has been received by the Prince of Wales:—

Brocket Hall, Hatfield, January 1st, 1905.

SIR,—I am very sorry to hear that the anonymous offer to contribute the necessary capital to provide one-third of the annual sum required, say, £14,000, to make the fixed income of the King's Hospital Fund up to £50,000 a year, has lapsed, in consequence of the inadequacy of the response to the appeal made by your Royal Highness now nearly a year ago.

Having for many years past taken a great interest in all that concerns the sick poor, and having from its foundation regarded the King's Fund as an ideal organisation for the distribution of the gifts and subscriptions of all those who, like myself, have no personal interest in any particular hospital or convalescent home, it is, therefore, a great pleasure to me to be able to send your Royal Highness, enclosed in this, an order for the delivery to your order, as President of the Fund, £100,000 Argentine Government Funding Bonds and £100,000 Buenos Ayres Water Works Bonds. These bonds yield an annual income of £11,000 a year, leaving £3,000 a year still to be provided for, affording a great opportunity for some one both willing and able to do a beneficent act in aid of the most deserving of all our charities.

I am sorry to see that the distribution this year is only £80,000, just a little over half the sum aimed at on the foundation of the Fund.

I sincerely hope the distribution will go on increasing from year to year until the £150,000 originally aimed at has been reached.

Believe me, Sir, your Royal Highness's most obedient servant,
MOUNT STEPHEN.

---

The King has sent the following letter to Lord Mount Stephen:—

Sandringham, Norfolk, January 2nd, 1905.

MY DEAR LORD MOUNT STEPHEN,—The Prince of Wales has just shown me your letter of yesterday's date.

As the founder of what is at present called "The King's Hospital Fund for London," which I originated to commemorate the late Queen's "Diamond Jubilee," I am anxious to lose no time in expressing to you my high appreciation of your magnificent donation.

I have not forgotten that it is the second large donation which you have so kindly given, and I have now every hope that, owing to your great liberality, my Fund will now be placed in the sound permanent position I had always hoped it would attain.

Believe me, very sincerely yours,
EDWARD R.

The Lord Mount Stephen, etc.

